# Rational Use of Benzodiazepines in Modern Healthcare: Evidence-Based Strategies

**DOI:** 10.3390/healthcare14101392

**Published:** 2026-05-19

**Authors:** Janko Samardžić, Neha Tandon, Milica Branković

**Affiliations:** Institute of Pharmacology, Clinical Pharmacology and Toxicology, Medical Faculty, University of Belgrade, 11000 Belgrade, Serbia; nehatandon@hotmail.com (N.T.); milica.radosavljevic@med.bg.ac.rs (M.B.)

**Keywords:** benzodiazepines, rational prescribing, dependence, insomnia, anxiety, geriatric pharmacology, precision medicine

## Abstract

Benzodiazepines (BZDs) are among the most widely prescribed psychotropic drugs in modern healthcare, primarily used as anxiolytics, hypnotics, anticonvulsants, and muscle relaxants. However, rising global consumption and prolonged use raise significant concerns about safety and dependence. Recent studies report that up to 35.8% of patients continue BZD therapy beyond three months, with long-term use observed in over 5% of the general population. These patterns highlight the need for evidence-based strategies to improve prescribing practices. BZDs are recommended primarily for short-term management of severe anxiety or transient insomnia, typically limited to 2–4 weeks. In anxiety disorders, SSRIs and SNRIs are first-line treatments. BZD use is particularly discouraged in older adults due to increased risks of cognitive impairment, falls, and dependence. Rational prescribing requires individualized assessment, minimal effective dosing, gradual withdrawal protocols, and patient education. Enhanced regulatory oversight and improved access to psychotherapy are essential for safer benzodiazepine use.

## 1. Introduction

The clinical adoption of benzodiazepines (BZDs) during the mid-20th century marked a turning point in sedative pharmacotherapy, largely replacing barbiturates due to their broader safety margin and reduced toxicity in overdose [1]. The synthesis of chlordiazepoxide in 1955 and the subsequent introduction of diazepam revolutionized psychopharmacology by providing agents with a wider therapeutic index, a lower risk of fatal overdose, and improved tolerability compared with barbiturates [1,2]. These characteristics quickly established BZDs as first-line medications for a broad range of neuropsychiatric conditions. Initially perceived as non-addictive and suitable for long-term use, BZDs were widely prescribed across medical specialties. However, increasing awareness of tolerance and dependence, as well as the risk of misuse and abuse, has restricted their use to a limited range of clinical indications [3].

Data from the United States show that between 1996 and 2013, the number of adults prescribed BZDs increased by 67%. This rise has been accompanied by growing concern regarding their adverse effects, particularly when combined with other psychotropic substances [4]. Consistent with these findings, BZDs remain among the most frequently prescribed psychotropic medications worldwide. Recent population-based studies indicate that between 5% and 10% of adults in many high-income countries receive at least one benzodiazepine (BZD) prescription annually, with a substantial proportion of patients continuing therapy beyond recommended durations [4,5]. Long-term use, commonly defined as treatment exceeding three months, has been reported in up to one third of patients, especially in older adults and individuals with comorbid psychiatric or somatic conditions [5].

Selective serotonin reuptake inhibitors (SSRIs) and serotonin–norepinephrine reuptake inhibitors (SNRIs) are now established as first-line therapies for most anxiety disorders [6]. At the same time, the risks associated with BZD use have become increasingly evident, particularly in vulnerable populations such as older adults, patients exposed to polypharmacy, and individuals with a history of substance use disorders [6,7]. Adverse effects, including cognitive impairment, increased risk of falls, motor vehicle accidents, and drug dependence, impose substantial clinical and social burdens. BZD use in geriatric populations is particularly discouraged due to age-related pharmacokinetic and pharmacodynamic changes that increase the risk of dependence, cognitive decline, and falls [7,8]. Rational BZD prescribing therefore requires careful patient selection, adherence to the minimal effective dose, implementation of gradual withdrawal protocols when appropriate, and continuous patient education [7,8,9].

This narrative review aims to critically evaluate the rational use of BZDs and summarize current evidence on strategies to improve safety and prescribing practices in modern healthcare.

## 2. Literature Search Strategy

A narrative literature search was conducted using PubMed, Scopus, and the Cochrane Library to identify relevant publications on BZD pharmacology, clinical indications, prescribing patterns, and safety concerns. Articles published primarily between 1994 and 2026 were considered. The search was conducted up to January 2026. The search strategy included combinations of keywords such as “benzodiazepines”, “rational prescribing”, “dependence”, “withdrawal”, “insomnia”, and “anxiety disorders”. Example search strings included: (“benzodiazepines” AND “rational prescribing”), (“benzodiazepines” AND “dependence” AND “withdrawal”), and (“benzodiazepines” AND “insomnia” OR “anxiety disorders”). Searches were limited primarily to English-language publications involving adult populations. Studies were selected based on their relevance to the clinical and pharmacological aspects of BZD use, with particular emphasis on recent publications, clinical guidelines, systematic reviews, and large observational studies. Additionally, relevant international clinical guidelines and consensus statements were consulted.

## 3. Pharmacology of Benzodiazepines

### 3.1. Mechanism of Action

BZDs exert their pharmacological effects by acting as positive allosteric modulators of the γ-aminobutyric acid type A (GABA_A_) receptor, the primary inhibitory neurotransmitter receptor in the central nervous system (CNS) [10]. The GABA_A_ receptor is a pentameric ligand-gated ion channel composed of five subunits, most commonly arranged as two α, two β, and one γ subunit. Two equivalent binding sites for GABA are located between the α and β subunits, whereas the BZD-binding site is situated between an α and a γ subunit. By binding to this site, BZDs enhance the affinity of the receptor for GABA, leading to an increased frequency of chloride channel opening [10,11]. This results in neuronal hyperpolarization and reduced excitability, thereby producing anxiolytic, sedative/hypnotic, anticonvulsant, and muscle-relaxant effects. Importantly, BZDs do not directly activate the GABA_A_ receptor in the absence of GABA, which contributes to their comparatively favorable safety profile relative to older sedative–hypnotics [10,11].

The clinical effects of BZDs are largely determined by their differential activity at distinct α subunits of the GABA_A_ receptor. Activation of α1 subunits is primarily associated with sedative, hypnotic, and amnestic effects, whereas modulation of α2 and α3 subunits mediates anxiolytic and muscle-relaxant actions [3,11]. In contrast, α5 subunit involvement has been linked to cognitive impairment and memory dysfunction. This subunit selectivity provides an explanation for both the therapeutic effects and adverse effects of BZD therapy and has stimulated interest in the development of selective ligands aimed at preserving anxiolytic efficacy while minimizing sedation and cognitive impairment [3,11,12]. Selective ligands at the BZD-binding site with a relative preference for GABA_A_ receptors containing the α1 subunit are commonly referred to as “Z-drugs,” reflecting the initial letter of their generic names (e.g., zolpidem and zopiclone) [13]. These agents are used primarily as hypnotics in clinical practice. Although structurally distinct from BZDs, Z-drugs share a similar mechanism of action, adverse effect profile, and pronounced hypnotic properties and therefore cannot be considered entirely separate from the BZD class [13,14].

### 3.2. Pharmacokinetics

BZDs are well absorbed following oral administration and can also be administered intravenously in acute conditions. They exhibit extensive plasma protein binding. Because of their high lipophilicity, many BZDs readily distribute into adipose tissue, where they may accumulate with repeated dosing. Their metabolism occurs predominantly in the liver via two major pathways: glucuronide conjugation and microsomal oxidation, followed by renal excretion [3]. Certain BZDs, including oxazepam and lorazepam, already possess a hydroxyl group and therefore undergo direct glucuronide conjugation without prior oxidative metabolism. As a result, these drugs are characterised by shorter elimination half-lives and a reduced potential for accumulation [3,15]. In contrast, most BZDs undergo hepatic oxidation or demethylation before conjugation, a process that frequently leads to the formation of active metabolites, such as desmethyldiazepam. This metabolic pathway is associated with prolonged elimination half-lives and an increased risk of drug accumulation, particularly during long-term use [15]. The pharmacokinetic properties of commonly prescribed BZDs are presented in Table 1.

### 3.3. Classification

According to the Anatomical Therapeutic Chemical (ATC) classification system, BZDs belong to the main group “N-Drugs acting on the nervous system,” within which they are further categorised as antiepileptics, anxiolytics, and hypnotics/sedatives. In addition to ATC classification, BZDs may be categorised according to their pharmacological potency (high vs. low) and duration of action. Based on elimination half-life, BZDs are commonly classified as long-acting (>48 h), intermediate-acting (24–48 h), short-acting (<24 h) [16], and ultra-short-acting (1–7 h) drugs (Table 2).

## 4. Indications, Contraindications and Use of BZDs

In modern psychiatric clinical practice, BZDs are indicated for the short-term management of panic disorder, generalised anxiety disorder, social anxiety disorder, and insomnia. In certain cases, they may be used as adjunctive therapy, such as in combination with SSRIs for obsessive–compulsive disorder (OCD) or alongside antipsychotics in the acute treatment of mania or severe agitation [17,18]. Beyond psychiatric indications, BZDs are considered first-line drugs in status epilepticus and are commonly used in alcohol withdrawal syndrome, especially in severe cases complicated by delirium tremens [19,20]. Additional indications include the treatment of acute dystonia and use as premedication in anesthesia [21].

Despite these clearly defined indications, BZDs are frequently prescribed in situations where evidence supporting their efficacy is limited, in patients with comorbidities that represent relative or absolute contraindications, or prior to adequate trials of recommended first- and second-line therapies [22]. BZDs should be used with caution in patients with major depressive disorder, suicidal ideation, bipolar disorder, psychotic disorders, or cognitive impairment, due to the risk of symptom exacerbation, dependence, and adverse cognitive effects. Their use is also contraindicated in individuals with current or past substance use disorders due to the high risk of abuse [23]. From a somatic perspective, BZDs are contraindicated in acute respiratory failure and respiratory depression, angle-closure glaucoma, severe renal insufficiency, porphyria, and myasthenia gravis [24]. In patients with chronic respiratory conditions such as chronic obstructive pulmonary disease (COPD) or obstructive sleep apnea, BZDs should be used with extreme caution and close clinical monitoring. Caution is also needed in obese patients, in whom increased adipose tissue may lead to drug accumulation and prolonged half-life. Special consideration is required in older adults [24].

Contemporary geriatric prescribing standards advise against routine BZD use in older adults. Namely, according to the American Geriatrics Society Beers Criteria, BZDs should generally be avoided in elderly patients, due to the increased risk of cognitive impairment, delirium, sedation, falls, fractures, and other adverse effects [25]. Therefore, BZD treatment should be limited to short-term use, generally not exceeding 2–4 weeks, in accordance with current clinical guidelines, as there is no convincing evidence supporting their long-term efficacy in the treatment of anxiety disorders or insomnia [26].

Although clear guideline recommendations restrict BZD use to short-term treatment, long-term and potentially inappropriate prescribing remains common in clinical practice. Epidemiological studies consistently report that a significant proportion of patients continue BZD therapy beyond recommended durations, with long-term use observed in up to one third of users in some populations [9]. This pattern is particularly pronounced among older adults and individuals with psychiatric or somatic comorbidities. A key contributor to long-term use is the gradual transition from short-term to maintenance therapy. What is initially prescribed for acute anxiety, insomnia, or situational distress may persist without regular reassessment of ongoing need, efficacy, or risk [27].

## 5. Rational Use in Modern Clinical Practice

### 5.1. Epidemiology and Patterns of Use

BZDs are among the most frequently prescribed psychotropic drugs worldwide [28]. Although the present review focuses on global prescribing patterns, country-specific pharmacoepidemiological studies can provide useful illustrative examples of BZD utilization trends. Serbia is presented as an illustrative example of national prescribing patterns due to the availability of detailed utilization data and the possibility of comparison with broader European and international trends. In Serbia, the use of psychotropic drugs has increased over the past decade, with anxiolytics such as bromazepam, diazepam, lorazepam, alprazolam, and prazepam showing the highest prevalence between 2014 and 2016 [28]. More recent pharmacoepidemiological studies demonstrate a continued rise in BZD consumption between 2018 and 2022, with levels consistently exceeding those reported in most European countries [29]. The COVID-19 pandemic further amplified this trend. Analyses of anxiolytic drug utilization in Serbia during 2019–2021 revealed a significant increase in BZD consumption, particularly bromazepam and alprazolam, coinciding with reduced access to mental health services and increased psychological distress. Notably, BZD use during this period remained approximately 2.5-fold higher than the European average. The highest consumption was observed in the elderly population, which is particularly vulnerable to BZD-associated adverse effects such as falls, fractures, and cognitive impairment [29].

A British study reported anxiolytic BZD use rates at 0.4% (age 15–44), 0.8% (age 45–65), and 1.9% (over 65), while hypnotic use increased from 0.3% to 1.4% and 5.2% across the same age groups, respectively [30]. Long-term use is also common: 35.8% of patients continue BZD therapy three months after initiation, 4.8% continue for up to eight years, and approximately 3% of the general population use BZDs long-term [31,32].

### 5.2. Practical Framework for Rational Benzodiazepine Prescribing

Rational BZD prescribing requires a structured clinical approach that balances potential therapeutic benefits with the well-documented risks of dependence, cognitive impairment, and misuse. A practical prescribing framework may assist clinicians in minimizing inappropriate use while ensuring effective symptom control [9,33]. First, the clinical indication should be clearly confirmed, ensuring that BZDs are used only when evidence-based indications are present, such as acute severe anxiety, panic attacks, status epilepticus, or alcohol withdrawal. Second, an individualized risk assessment should be performed, considering patient age, comorbidities, history of substance use disorders, and concurrent medications that may increase the risk of adverse effects. Third, treatment duration should be limited to the shortest period necessary, typically not exceeding two to four weeks. Patients should be informed at the initiation of therapy that BZDs are intended for short-term use. In addition, shared decision-making plays a crucial role in optimising treatment outcomes. Clinicians should engage patients in discussions regarding the expected benefits and risks of therapy, duration of use, and potential withdrawal symptoms. Involving patients in planning dose reduction and discontinuation strategies may improve adherence and facilitate successful deprescribing. Moreover, regular clinical reassessment is essential to evaluate treatment efficacy, adverse effects, and the ongoing need for therapy. Finally, clinicians should actively identify deprescribing triggers, such as symptom improvement, development of tolerance, adverse cognitive effects, or the availability of safer alternative treatments [34,35].

### 5.3. Use in Anxiety Disorders

Given the delayed onset of antidepressant effects, which may require up to six weeks, BZDs may be used to manage acute exacerbations of generalised anxiety disorder. However, their use should be carefully limited to situations where rapid symptom control is required, such as acute anxiety states or severe functional impairment, particularly during the initiation phase of antidepressant therapy. In such cases, BZDs may serve as a short-term adjunct while awaiting the therapeutic effect of first-line treatments [20]. In comparison to BZDs, which provide rapid symptomatic relief, SSRIs and SNRIs have a more favorable long-term safety and tolerability profile but are associated with a delayed onset of action and may initially exacerbate anxiety symptoms in some patients. Therefore, treatment selection should be individualized, taking into account symptom severity, need for rapid relief, comorbidities, risk of dependence, and patient preference. Evidence also supports the efficacy of BZDs in treating symptoms of social anxiety and panic disorder. In generalised anxiety, low-potency BZDs are typically effective, whereas high-potency agents are more suitable for panic attacks and episodic anxiety [20]. Despite clear guideline recommendations favoring SSRIs and SNRIs as first-line therapies, BZDs remain widely used in clinical practice, reflecting a gap between evidence-based recommendations and real-world prescribing patterns. This discrepancy likely reflects the complex interaction of patient expectations, limited access to psychotherapy, clinician prescribing habits, healthcare system constraints, and the rapid symptomatic relief provided by BZDs. At the same time, certain patients may maintain clinical stability during prolonged BZD therapy without substantial dose escalation, reflecting the ongoing debate regarding the balance between therapeutic benefit and long-term risk [26,30,36].

Comorbid depression frequently complicates anxiety disorders and poses a significant therapeutic challenge. Overlapping symptoms such as negative thoughts, insomnia, irritability, fatigue, and suicidal ideation may obscure the diagnosis and reduce caution in BZD prescribing. Several studies have reported an association between BZD use and depression, as well as an increased risk of suicide. However, these findings are primarily based on observational data and may be influenced by confounding factors such as underlying psychiatric illness [24]. Long-term BZD use can worsen depressive symptoms and induce neuroadaptive changes, including reduced GABA concentrations, impaired monoaminergic function, decreased neurogenesis, and cognitive decline. These changes may reduce antidepressant efficacy [37]. While anxiolytic and hypnotic effects often diminish over time due to tolerance, persistent depressive symptoms and impulsivity may be misinterpreted as disease progression rather than consequences of long-term BZD use [24,38].

### 5.4. Use in Insomnia

In addition to anxiety disorders, BZDs are frequently prescribed for insomnia. Although these agents shorten sleep onset and extend overall sleep time, they modify physiological sleep patterns by suppressing slow-wave and REM phases, potentially compromising restorative sleep quality [38]. These changes may persist even after discontinuation. Consequently, BZDs are indicated only for transient or short-term insomnia, with therapy typically limited to 2–4 weeks [36]. Chronic insomnia requires careful evaluation, and hypnotics should be prescribed at the lowest effective dose for short-term use. According to the British National Formulary, BZDs should be reserved for long-term, disabling insomnia that causes significant distress when first-line treatments fail [30,36]. A key concern with BZDs as hypnotics is the rapid development of tolerance, which can prompt dose escalation and prolonged use. Withdrawal is frequently associated with rebound insomnia, characterised by poorer sleep quality, delayed sleep onset, and frequent awakenings, which may persist for several weeks [36].

In clinical practice, non-benzodiazepine hypnotics, commonly referred to as Z-drugs (e.g., zolpidem and zopiclone), are frequently used as pharmacological options for the treatment of insomnia. Because of their similar clinical effects and safety considerations, these agents are often considered alongside BZDs when making therapeutic decisions for insomnia. However, evidence suggests that Z-drugs share many of the same risks as BZDs, including tolerance, dependence, cognitive impairment, and increased risk of falls, particularly with prolonged use. Consequently, current clinical guidelines recommend limiting their use to short-term treatment [13,14]. Long-term use of BZDs and Z-drugs for chronic insomnia is generally discouraged due to the risks of tolerance, dependence, cognitive impairment, and rebound insomnia [14,27,36]. Non-pharmacological interventions, including sleep hygiene education and cognitive–behavioral therapy, represent first-line treatment for insomnia. If pharmacological support is necessary, low-dose doxepin may be considered as an option [27].

## 6. Safety Concerns and Adverse Effects

### 6.1. Dependence and Withdrawal

One of the most significant limitations of BZD therapy is the risk of tolerance, dependence, and withdrawal, particularly with prolonged use. Tolerance develops as a result of neuroadaptive changes within the GABAergic system, including receptor downregulation and altered subunit composition, leading to a gradual reduction in therapeutic efficacy. These neuroadaptive changes result in reduced inhibitory GABAergic tone and a relative increase in excitatory neurotransmission, leading to neuronal hyperexcitability, which underlies the development of withdrawal symptoms. In addition, alterations in other neurotransmitter systems, including glutamatergic and monoaminergic pathways, may further contribute to the complexity of withdrawal phenomena. Therefore, patients may require higher doses, further increasing the risk of adverse effects [39].

Abrupt discontinuation of BZDs can precipitate withdrawal symptoms, which commonly include rebound anxiety and insomnia. Additional manifestations may involve irritability, tremor, autonomic hyperactivity, perceptual disturbances, and, in severe cases, seizures [24,39]. BZDs may also cause paradoxical excitation, a disinhibitory effect that can manifest as increased anxiety, hyperactivity, and aggression, particularly in vulnerable populations such as children and adolescents [39,40].

Management of BZD withdrawal requires a gradual and individualized tapering strategy in order to minimize withdrawal symptoms and reduce the risk of relapse. Deprescribing should be prioritized in patients receiving long-term therapy, high doses, or those at increased risk of adverse effects, including older adults and individuals with comorbid psychiatric or substance use disorders. Rebound insomnia and anxiety are common during withdrawal and can be managed by gradual tapering, patient education, reassurance, and, when needed, short-term supportive interventions. Current clinical recommendations suggest progressive dose reduction, typically by approximately 5–10% of the daily dose every 2–4 weeks, depending on the duration of prior treatment, the specific BZD used, and patient tolerance [9,33]. For example, in a patient receiving 2 mg of lorazepam daily, the dose may be gradually reduced by 0.25–0.5 mg every 2–4 weeks, with slower reductions at lower doses depending on patient tolerance. In individuals receiving long-term therapy or higher doses, tapering schedules should be further individualized and may extend over several months to minimise withdrawal symptoms and reduce the risk of relapse [33].

In some cases, substitution with a long-acting BZD, most commonly diazepam, may facilitate safer discontinuation [41]. For example, approximately 0.5 mg of alprazolam is considered equivalent to 10 mg of diazepam, which may facilitate conversion to a long-acting agent during tapering. Similarly, 1 mg of lorazepam or 0.5 mg of clonazepam is generally considered approximately equivalent to 10 mg of diazepam (Table 3) [42]. Due to its long elimination half-life and active metabolites, diazepam provides more stable plasma concentrations and may reduce the severity of withdrawal symptoms during tapering.

These equivalence values are approximate and should be individualized based on clinical response, particularly in older adults and patients with hepatic impairment. Following conversion to an equivalent diazepam dose, gradual dose reduction can be implemented over several weeks or months depending on clinical response [33,41]. Successful BZD discontinuation often requires a multidisciplinary approach. Pharmacists can play an important role in monitoring drug interactions, counseling patients, and supporting adherence to tapering regimens [43].

### 6.2. Cognitive and Psychomotor Effects

Short-term and long-term use of BZDs is associated with impairments in attention, processing speed, and memory, particularly anterograde amnesia [44]. These effects are more pronounced with long-acting drugs, those with active metabolites, and during long-term therapy. Long-term BZD users may become more sensitive to these effects with advancing age, likely due to neuronal loss and reduced receptor reserve. Clinically, this increased sensitivity may lead to various cognitive symptoms that can mimic neurodegenerative disorders, a phenomenon often referred to as BZD-associated “pseudodementia” [30]. Although potentially reversible upon drug discontinuation, this condition poses significant diagnostic challenges and may result in unnecessary investigations or misdiagnosis, particularly in older adults.

Older adults are especially vulnerable to the adverse effects of BZDs due to age-related pharmacokinetic and pharmacodynamic changes, including reduced hepatic clearance, increased volume of distribution for lipophilic drugs, and enhanced CNS sensitivity. In this population, BZD use is strongly associated with an increased risk of falls and fractures, as well as the development of delirium, particularly during acute illness or hospitalization [30,45]. Epidemiological studies have shown that BZD use is associated with a significantly increased risk of falls and fractures in older adults, with estimates suggesting up to a 1.5–2-fold increase in risk compared to non-users [46]. In addition to cognitive impairment, BZDs reduce reaction time and impair coordination, significantly increasing the risk of motor vehicle accidents and occupational injuries [47]. Epidemiological studies have consistently demonstrated an association between BZD use and increased accident risk, comparable to moderate alcohol intoxication. In the United States, over 10% of women and 6% of men aged 65–80 receive BZD prescriptions annually, with nearly one-third using them for more than 120 days [45]. Z-drugs carry comparable risks and should not be regarded as safer alternatives [27].

### 6.3. Drug Interactions and Polypharmacy

Polypharmacy represents an important factor contributing to BZD-related adverse effects [9,33,48]. Concomitant use of BZDs with other CNS depressants significantly increases the risk of excessive sedation, respiratory depression, and overdose. Particularly concerning is the co-prescription of BZDs with opioids, which has been strongly associated with increased mortality due to additive respiratory depressant effects. In addition, co-prescription with opioids has been associated with a substantial increase in overdose-related mortality, with BZDs involved in a significant proportion of fatal opioid overdoses [49]. For this reason, several clinical guidelines recommend avoiding this combination whenever possible or using the lowest effective doses with careful monitoring when co-administration cannot be avoided [33,50,51].

Similar risks have been reported with the concurrent use of BZDs and gabapentinoids (e.g., gabapentin, pregabalin), which may potentiate CNS depression and further increase the risk of respiratory complications, especially in older adults and patients with underlying respiratory disorders [52]. Alcohol consumption also significantly enhances the sedative effects of BZDs and may lead to impaired psychomotor performance, increased risk of accidents, and potentially life-threatening respiratory depression [53]. In addition, BZDs are frequently prescribed together with antipsychotics and other psychotropic medications. While such combinations may sometimes be clinically justified, particularly in the management of acute agitation, careful assessment of the risk–benefit ratio is required due to the potential for cumulative sedation, cognitive impairment, and increased fall risk [54,55].

### 6.4. Risk Groups and Prevention Strategies

To identify individuals at risk and implement preventive measures, BZD users can be categorized based on clinical characteristics and patterns of use. Patients with frequent dysphoria and chronic sleep disturbances are associated with higher rates of misuse [38]. Prescribing to this population should be avoided due to cognitive adverse effects that impair functional adaptation [38].

Stricter prescription control represents one strategy for addressing BZD misuse. Regulatory policies and prescription monitoring programs play an important role in reducing inappropriate BZD use. Prescription drug monitoring programs (PDMPs) can help identify high-risk prescribing patterns, including long-term use, high-dose therapy, and clinically significant drug interactions. However, their effectiveness depends on consistent implementation, clinician engagement, and integration into clinical practice [56]. Moreover, patient education regarding the risks of long-term use is essential for preventing dependence. Therapy should rarely exceed 2–4 weeks, dose escalation should be avoided, and intermittent rather than continuous use may be more appropriate for insomnia management. Careful reevaluation, appropriate dosing, and timely discontinuation are critical preventive measures. Particular attention should be paid to high-risk groups, including patients with a history of alcohol abuse, chronic insomnia, personality disorders, or dysthymia [57].

General practitioners prescribe the majority of BZDs, primarily due to frequent contact with elderly patients. Challenges to reducing overprescription include underestimation of adverse effects, the belief that benefits outweigh risks, limited time for supervised tapering, fear of damaging patient–physician trust, and reluctance to modify another clinician’s prescription [27]. Fear of withdrawal symptoms, patient resistance to discontinuation, and limited availability of non-pharmacological treatment options further contribute to chronic BZD use in clinical practice [27,34,57]. Additional issues include limited access to psychotherapists and psychiatrists, particularly during withdrawal, and overuse of psychotropic drugs in nursing homes. These institutions should therefore also be targeted for preventive interventions.

## 7. Conclusions

BZDs remain widely used in clinical practice, reflecting both their therapeutic effectiveness and the challenges associated with their appropriate use. While concerns regarding potential overuse and misuse are well documented, BZDs continue to play an important role in specific conditions where rapid symptom control is required. Independent studies are therefore needed to objectively assess factors contributing to BZD overuse and to reevaluate current pharmacotherapeutic approaches and prescribing patterns. Enhanced communication between different levels of healthcare and broader access to updated clinical guidelines can help reduce inappropriate prescribing. From a clinical perspective, BZD prescribing should be restricted to clearly defined indications, at the lowest effective dose and for the shortest possible duration, with regular reassessment and early planning of discontinuation strategies. Particular attention should be given to high-risk populations, including older adults and patients with a history of substance use disorders. At the policy level, implementation of prescription monitoring programs, adherence to evidence-based guidelines, and improved access to non-pharmacological therapies are essential to reduce inappropriate use. Finally, patient and public education play a crucial role in promoting informed use, improving adherence, and facilitating the safe discontinuation of long-term BZD therapy.

### Limitations of the Review

This review has several limitations that should be considered when interpreting the findings. First, as a narrative review, it does not follow a systematic methodology for study selection, which may introduce selection bias and limit reproducibility. Second, although efforts were made to include recent and relevant literature, the available evidence is heterogeneous, with variations in study populations and outcome measures, which may affect the consistency of conclusions. In addition, the interpretation of BZD-related outcomes remains methodologically challenging due to differences in prescribing practices, duration of exposure, psychiatric comorbidities, and concurrent medication use across studies. The distinction between appropriate therapeutic use, dependence, and misuse may also vary depending on clinical, social, and healthcare contexts. Finally, while this review emphasizes rational prescribing strategies, their practical implementation may vary depending on healthcare infrastructure, availability of non-pharmacological treatments, and clinician experience. Future research should further explore the complex clinical and psychosocial factors contributing to prolonged BZD use and evaluate strategies aimed at improving deprescribing practices in different healthcare settings.

## Figures and Tables

**Table 1:** Pharmacokinetic properties of commonly prescribed BZDs.

Drug	Tmax (h)	Plasma Protein Binding (%)	Average Elimination Half-Life (h)
Alprazolam	1–2	80	6–27
Bromazepam	1–4	70	8–19
Diazepam	1–2	97	20–100
Lorazepam	1–6	75	10–20
Clonazepam	1–4	84	20–60
Midazolam	1	95	1.5–2.5

**Table 2:** Classification of the most commonly prescribed BZDs.

Drug	ATC Classification	Potency	Duration of Action
Alprazolam	Anxiolytic	High-potency	Short-acting
Bromazepam	Anxiolytic	Medium-potency	Intermediate duration
Diazepam	Anxiolytic	Medium-potency	Long-acting
Lorazepam	Anxiolytic	High-potency	Short-acting
Clonazepam	Antiepileptic	High-potency	Long-acting
Midazolam	Hypnotic, sedative	High-potency	Ultra-short-acting
Oxazepam	Anxiolytic	Low-potency	Short-acting

**Table 3:** Approximate oral BZD dose equivalents relative to diazepam 10 mg.

Drug	Approximate Equivalent Dose to Diazepam 10 mg
Alprazolam	0.5
Bromazepam	6
Clobazam	20
Clonazepam	0.5
Lorazepam	1
Oxazepam	20
Flunitrazepam	0.5–1
Nitrazepam	5–10
Temazepam	20

## Data Availability

No new data were created or analysed in this study. Data sharing is not applicable to this article.
